# A Small Intestinal Stromal Tumor Detection Method Based on an Attention Balance Feature Pyramid

**DOI:** 10.3390/s23249723

**Published:** 2023-12-09

**Authors:** Fei Xie, Jianguo Ju, Tongtong Zhang, Hexu Wang, Jindong Liu, Juan Wang, Yang Zhou, Xuesong Zhao

**Affiliations:** 1 Xi’an Key Laboratory of Human–Machine Integration and Control Technology for Intelligent Rehabilitation, Xijing University, Xi’an 710123, China; xiefei@xijing.edu.cn (F.X.); wangjuan@xijing.edu.cn (J.W.); 2 School of AOAIR, Xidian University, Xi’an 710075, China; 3 School of Information Science and Technology, Northwest University, Xi’an 710126, China; zhangtongtong@stumail.nwu.edu.cn (T.Z.); amosliu@stumail.nwu.edu.cn (J.L.); 201831967@stumail.nwu.edu.cn (Y.Z.); 4 Departments of Radiology, Ruijin Hospital, Shanghai Jiao Tong University School of Medicine, Shanghai 200025, China

**Keywords:** small intestinal stromal tumor, object detection, attention balanced feature pyramid, deep neural network

## Abstract

Small intestinal stromal tumor (SIST) is a common gastrointestinal tumor. Currently, SIST diagnosis relies on clinical radiologists reviewing CT images from medical imaging sensors. However, this method is inefficient and greatly affected by subjective factors. The automatic detection method for stromal tumors based on computer vision technology can better solve these problems. However, in CT images, SIST have different shapes and sizes, blurred edge texture, and little difference from surrounding normal tissues, which to a large extent challenges the use of computer vision technology for the automatic detection of stromal tumors. Furthermore, there are the following issues in the research on the detection and recognition of SIST. After analyzing mainstream target detection models on SIST data, it was discovered that there is an imbalance in the features at different levels during the feature fusion stage of the network model. Therefore, this paper proposes an algorithm, based on the attention balance feature pyramid (ABFP), for detecting SIST with unbalanced feature fusion in the target detection model. By combining weighted multi-level feature maps from the backbone network, the algorithm creates a balanced semantic feature map. Spatial attention and channel attention modules are then introduced to enhance this map. In the feature fusion stage, the algorithm scales the enhanced balanced semantic feature map to the size of each level feature map and enhances the original feature information with the original feature map, effectively addressing the imbalance between deep and shallow features. Consequently, the SIST detection model’s detection performance is significantly improved, and the method is highly versatile. Experimental results show that the ABFP method can enhance traditional target detection methods, and is compatible with various models and feature fusion strategies.

## 1. Introduction

The incidence rate of small intestinal diseases is high and gradually increasing. However, the onset of small intestinal diseases is slow, and the symptoms are not specific, which makes it difficult to diagnose as compared to other gastrointestinal diseases. Moreover, the diagnosis of small intestinal digestive system diseases is challenging, and the lack of case data and public datasets makes it harder to conduct research on auxiliary diagnoses of small intestinal tissue diseases. As a result, there are hardly any research findings on the subject.

At present, the diagnosis of SIST mainly depends on multi-slice spiral CT and high-field MRI equipment [1,2]. At present, although enhanced scanning imaging technology has been used in the process of CT scanning, doctors mainly rely on their own clinical diagnosis experience in the diagnosis of SIST. Because SIST exists in a variety of shapes, sizes, and densities, some SIST will change their position with the small intestine peristalsis, which is difficult to observe and distinguish, all of which pose great challenges to doctors in diagnosis. However, few doctors with rich experience in the diagnosis of SIST, especially in areas with relatively poor medical resources, make it difficult to detect SIST in time. This will lead to many patients with early stromal tumors not being able to receive timely treatment, delaying the best time for treatment. On the other hand, different doctors have different diagnosis experiences and personal subjective analyses and judgments during diagnosis, resulting in different diagnosis results of SIST, which will also give different treatment plans. If an appropriate treatment plan cannot be objectively given, the condition will be delayed or even worsened.

**Figure 1:**
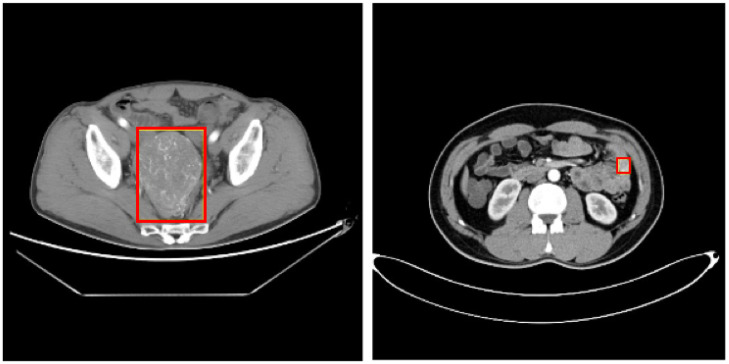
CT image of SIST (the red surrounding box is the focus area).

In the traditional diagnosis of SIST, doctors judge whether the patient has SIST by observing the different stages of CT/MR images of the patient, namely the plain scan stage, arterial enhancement stage and portal enhancement stage, combined with pathology. In this paper, we use the image data labeled by professional doctors, and use the theory of deep learning and strong supervised learning to train the network model to detect the area of SIST, and carry out the auxiliary diagnosis of SIST. The characteristics of SIST are complex and diverse, as shown in Figure 1. Their shape and size differ greatly, and the boundary is not obvious. The difference between SIST and surrounding normal tissues is small, and the gray values of the two are close. This poses a huge challenge for the detection of SIST, which needs to solve the problem of focus edge similarity, avoid the interference of normal tissue on the recognition of stromal tumors, and design an optimization network for the complex features of stromal tumors, so as to achieve accurate detection of the focus area of stromal tumors in small intestinal medical images.

Aiming at the problem of unbalanced feature fusion of SIST data in the target detection model, this paper proposes the ABFP algorithm. First, the multi-level feature map extracted by the backbone network is scaled to the resolution of the middle layer, and then the scaled multi-level feature map is weighted and fused to obtain the balanced semantic feature map. Then, the balanced semantic feature map is refined and improved. This paper uses the channel attention and spatial attention mechanism to refine and improve the balanced semantic feature map to obtain the enhanced balanced semantic feature map. Finally, the enhanced balanced semantic feature map is re-scaled back to the original size of the feature map of each layer and added to the original feature map to enhance the original feature information, so as to improve the feature extraction ability of the SIST detection model and improve the detection accuracy.

To sum up, the main contributions of this paper are as follows: (1) To address the issue of feature imbalance during the feature fusion process, the Balanced Feature Pyramid algorithm is proposed. It aims to improve feature imbalance by balancing semantic feature maps. (2) By introducing channel attention modules and spatial attention modules into the Balanced Feature Pyramid, it enhances balanced semantic feature maps, ultimately improving the model’s detection performance. (3) The experimental results show that the ABFP method can improve the traditional target detection methods and can be compatible with different network models and feature fusion strategies.

## 2. Related Work

SIST is one of the common primary tumors of the gastrointestinal tract. The diagnosis of a stromal tumor mainly depends on the observation of CT and MRI images of patients by imaging doctors. At present, deep learning, computer vision and other methods have not been applied to the detection and recognition of SIST in image data. With the development of computer vision technology, computer vision technology has been used to assist in the diagnosis of other tumors or related focus areas.

In recent years, with the continuous maturity of image processing and deep learning technology, computer-aided diagnosis can help pathologists make more objective and effective diagnoses. The focus detection task in different medical image data has always been an important work in clinical work. The location in medical imaging usually needs to analyze the three-dimensional coordinate space of the target. Yang et al. [3] processed three sets of independent 2D magnetic resonance slices to detect the surface of the distal femur through the common three-dimensional convolution neural network. The intersection of the three two-dimensional slices with the highest classification probability output is the detected 3D position. DeVos et al. [4] further identified a 3D rectangular frame after converting a 2D slice into a 3D CT image, so as to find the region of interest (ROI) around the anatomical region. The pre-trained convolutional neural network architecture is applied to the same target as RBM [5,6], thus overcoming the lack of data and learning better feature representation. Payer et al. [7] proposed to use CNN to directly regression the key points. They used the coordinate graph as the true value, and the network was directly trained to predict the coordinate graph. Ghesu et al. [8] used reinforcement learning to identify key points, and showed amazing results in the processing tasks of 2D cardiac MRI images, ultrasound images (US), and 3D head/neck images. Rehman et al. [9] proposed 3D convolutional neural network (CNN) architecture for microscopic brain tumor detection and classification, a pre-trained CNN model is used for feature extraction, and the selected best features are validated through a feed-forward neural network for final classification. Ghesu et al. [10] proposed a sparse adaptive neural network driven by edge learning to solve the data complexity problem of transesophageal three-dimensional echocardiography in detecting aortic valves. A convolution neural network is also used to locate scanning planes or key slices in 3D medical image sequences. Baumgartner et al. [11] trained multiple convolutional networks to detect the position of twelve standard scanning planes in the second trimester of pregnancy. Furthermore, RNN, especially LSTM, is also used to process the timing information in medical video [12]. Gyawali P. K. et al. [13] showed the effect of linear mixing of input space and potential space on neural network data on semi-supervised learning in the classification task of thoracic diseases and skin diseases. Roth et al. [14] used a large number of axial CT images to train the deep convolution neural network to classify the anatomy of organs or specific parts of the body, including the neck, lungs, liver, pelvis and legs. Then, many other DNN-based methods are proposed for organ detection and recognition [15]. As long as an organ is correctly labeled, the corresponding section is considered to be correct.

Using deep learning to detect objects or damaged areas has also achieved good results. Detection of tissues, organs, or damaged areas is the core step of diagnosis. Usually, the task can be decomposed into the task of locating and classifying the lesion area in the whole image space. In addition, a multi-stream convolution neural network is used to process context information and 3D information fusion, among which the works of Barbu et al. [16] and Agnes et al. [12] are representative. Teramoto et al. [17] fused CT and PET data based on a multi-channel convolution neural network to detect pulmonary nodules. Continuing to use anatomical structure, Liu et al. [18] proposed a multi-organ localization method based on a 3D convolutional regression network. It can use coarse-to-fine approaching towards the target organs iteratively through learning the anatomy features. Udupa et al. [19] established a hybrid intelligence system that integrates the experience of human experts and the powerful learning ability from deep learning to perform organ localization and segmentation. Finally, in the work of generating training data annotation, which is of the same importance as object classification, Wang et al. [20] proposed a multi-task intracerebral hemorrhage detection network MMTNet, which encodes region-related features through a two-branch network, and then inputs these features into a multi-task classifier to predict regional intracerebral hemorrhage. Ju et al. [21] employed deep convolutional neural networks in combination with their proposed spatial visual cue fusion and active localization offset module to perform the segmentation of the pancreas and pancreatic tumors. Yang et al. [22] introduced a self-training-based semi-supervised detection method, incorporating an improved Faster R-CNN for the detection of gastrointestinal stromal tumors.

At present, there has been a lot of research work at home and abroad on the use of artificial intelligence technology for medical auxiliary diagnosis [23,24], especially the research on the inspection, recognition, and analysis of organs and tissues or focus areas in medical images based on computer vision technology, which has attracted more and more attention in the field of artificial intelligence and medical imaging. However, the research on detection and recognition of SIST has the following problems: after analyzing the mainstream target detection model on the SIST data, it was found that the features of different levels in the feature fusion stage of the network model are unbalanced. Therefore, this paper proposes the ABFP algorithm to solve the unbalanced feature fusion problem and improve the model detection performance.

## 3. The Proposed Method

SIST is one of the primary gastrointestinal tumors in the small intestine. In CT images, small intestinal stromal tumors vary in shape and size, and are difficult to accurately distinguish from surrounding normal tissues and organs. In view of the complex imaging manifestations of SIST, this section designs a detection method for SIST based on strong supervised learning theory. Through observing a large number of detection results, we found that the reason for these phenomena was that the feature information of normal tissues and organs in the image affected the feature recognition of the target detection model for the focus area of stromal tumors, and the unbalanced fusion of deep and shallow features extracted from the backbone feature network affected the recognition and detection ability of the detection model. In order to achieve accurate detection of SIST, solve the imbalance between deep and shallow features of the model, screen out important information in the network model training, and improve the feature extraction ability of the detection model, this section will introduce the BFP network algorithm for the detection model, which enhances the original feature map through the integrated balanced semantic feature map. In this way, each feature layer in the feature pyramid can obtain the same information from other layers, so as to balance the information flow and make the features more distinctive. In addition, this section introduces the channel attention module and the spatial attention module for the BFP network, which, respectively, consider the important information in the channel and space, which can enhance the balanced semantic feature map in the BFP, help the detection model to focus on the more important channel and region information, and reduce the adverse effects of normal tissues and organs on network training. The experimental results indicate that the proposed Attention-Based Balanced Feature Pyramid method in this section can enhance traditional object detection methods and is compatible with various network models and feature fusion strategies.

### 3.1. Balanced Feature Pyramid

With the rapid development of theoretical research on target detection in computer vision in recent years, many detection models have been designed and developed at home and abroad, such as Faster RCNN [25], RetinaNet [26], Cascade RCNN [27] and RepPoints [28]. These model frameworks have obvious differences in network architecture, which can be divided into single-stage detection, two-stage detection, and “1.5”-stage detection framework with one classification and two regression, and can also be divided into anchor-base and anchor-free detection methods, but most of these model frameworks follow a general training process; that is, sampling the region, extracting feature information from it, and then jointly identifying the category and improving the location information under the guidance of the multi-task target function. In the SIST detection task proposed in this paper, the performance of target detection model training depends on whether the extracted visual features are fully utilized.

Traditional backbone feature extraction networks, such as AlexNet, VGG, ResNet, ResNeXt and other deep high-level features, have more semantic information, while shallow low-level features have more content description information. The feature pyramid generated by multi-level features has a great impact on detection performance. However, the sequence method in the mainstream FPN or PAFPN methods pays more attention to the information of adjacent feature layers in the feature fusion process, and less attention to the information of non-adjacent layers. After each feature fusion, the semantic information of non-adjacent layers will be weakened. In order to solve this problem, this paper uses the BFP and the integrated balanced semantic feature map to enhance the original features, so that each feature layer in the feature pyramid can obtain equal information to each other, thus balancing the feature information.

**Figure 2:**
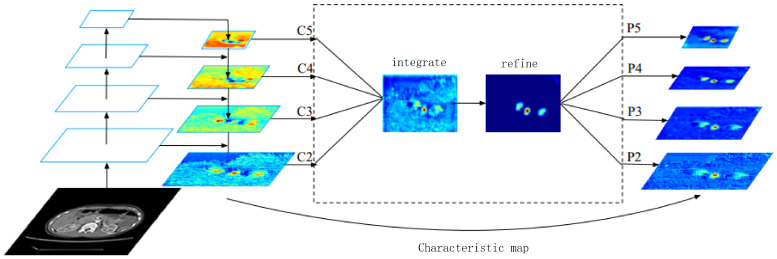
The basic structure diagram of BFP.

Different from the above method of integrating multi-level features using the horizontal connection, the main idea of BFP is to use the same deeply integrated balanced semantic feature map to enhance the original multi-level features. The model structure is shown in Figure 2. The model includes four parts, namely rescaling, integrating, refining, and enhancing.

In the process of obtaining balanced semantic features, the feature map with a resolution of *r* is defined as $C_{r}$. The number of multi-layer feature maps is expressed as *N*, where the lowest and highest resolution is recorded as $r_{min}$ and $r_{max}$. In Figure 2, $C_{2}$ has the highest resolution.

In order to fuse the multi-level feature map and retain its semantic information, we first adjust the multi-level feature map {$C_{2},C_{3},C_{4},C_{5}$} to the middle size. Then, interpolation and maximum pooling are performed, respectively. The feature maps at all levels are rescaled to the same size, and the balanced semantic feature map is obtained by adding and averaging. The calculation formula is shown in Equation (1):

(1) $$C=\frac{1}{N}\sum_{r=r_{min}}^{r_{max}}C_{r}$$

Then, use the same but opposite process to rescale the original feature map size to enhance the original feature information. In this process, features of each resolution level can obtain the same information from other layers, and this process does not contain other redundant parameters.

In terms of refining and improving the balanced semantic features, the more refined balanced semantic features are more discriminative. To this end, we refined and improved the features by adding attention modules. Refined and improved operations help us enhance the integration function and further improve the results. Then, output the corresponding {$P_{2},P_{3},P_{4},P_{5}$}, which is used in the target detection process after FPN.

### 3.2. Attention Mechanism Module

For the features of channel dimensions, the traditional convolution kernel is to fuse the features of all channels, so that the weight of each channel dimension is the same. However, the contribution of the feature map of each channel to feature extraction and final target detection is different. The idea based on the channel attention mechanism is to assign different weights to each channel to represent the importance of each channel. The greater the weight, the more important the characteristics of the channel are. The idea based on the spatial attention mechanism is to assign values to the importance of different positions of the feature map of each channel, enhance the features of important regions, and suppress the features of unimportant regions. This paper refines the balanced semantic features by combining channel attention and spatial attention.

**Figure 3:**
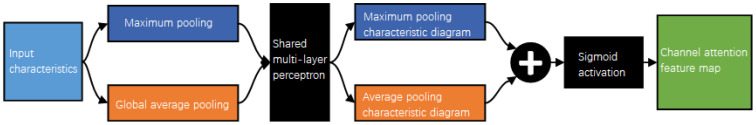
Model diagram of channel attention mechanism module.

The channel attention module gives weight according to the importance of the characteristic map of different channel dimensions. The input feature map is passed through AvgPool and MaxPool, respectively, and then the features after and are input into the shared network composed of multi-layer perceptron (MLP), respectively, and the output features are added and sigmoid activated to generate the final channel attention feature map $M_{c}\in R^{C\times 1\times 1}$; that is, the dimension is C×1×1. The calculation expressions are shown in Equations (2) and (3):

(2) $$M_{c}\left(F\right)=\sigma \left(MLP\left(AvgPool\left(F\right)\right)+MLP\left(MaxPool\left(F\right)\right)\right)$$

(3) $$M_{c}\left(F\right)=\sigma \left(W_{1}\left(W_{0}\left(F_{avg}^{c}\right)\right)+W_{1}\left(W_{0}\left(F_{max}^{c}\right)\right)\right)$$

where σ is sigmoid operation, *F* is input characteristic graph, AvgPool and MaxPool are average pooling and maximum pooling, respectively, $F_{avg}^{c}$ and $F_{max}^{c}$ are the features after average pooling and maximum pooling, $W_{0}$ and $W_{1}$ are the weight of MLP, where *r* is the reduction rate and *C* is the number of channels. Finally, perform the Relu operation as shown in Figure 3.

**Figure 4:**
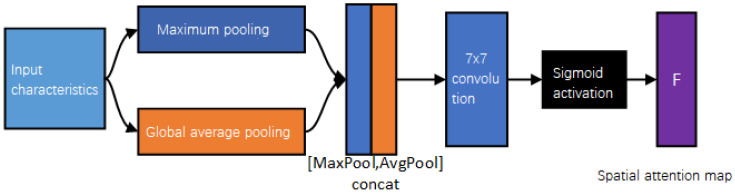
Model diagram of spatial attention mechanism module.

The spatial attention module determines the importance of different regions according to the correlation of the spatial position of the feature map in each channel, and enhances or suppresses the regions. Perform AvgPool and MaxPool operations on the input features, and perform Concat operations on the two results based on the channel, and then perform 7×7 convolution operation, and finally carry out activation processing to obtain the spatial attention feature map $M_{S}\in R\ldots \ldots 1\times H\times W$; that is, the dimension is H×W. The calculation is shown in Equations (4) and (5):

(4) $$M_{S}\left(F\right)=\sigma \left(f^{7\times 7}\left(\left[AvgPool\left(F\right);MaxPool\left(F\right)\right]\right)\right)$$

(5) $$M_{S}\left(F\right)=\sigma \left(f^{7\times 7}\left(\left[F_{Avg}^{s};F_{Max}^{s}\right]\right)\right)$$

where $f^{7\times 7}$ is a convolution operation, the filter size is 7×7, and $F_{Avg}^{s}$ and $F_{Max}^{s}$ are the characteristics after average pooling and maximum pooling, as shown in Figure 4.

### 3.3. Attention Balance Feature Pyramid Abfp

**Figure 5:**
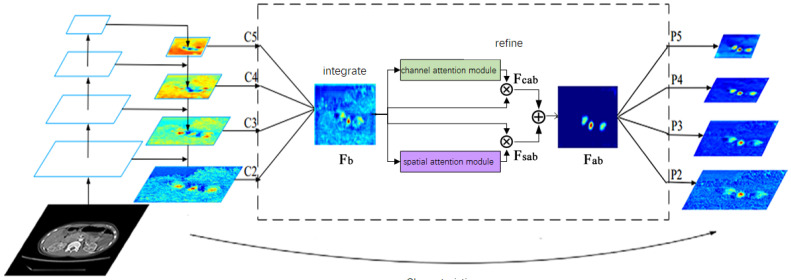
Structure diagram of ABFP.

In this paper, the ABFP is used to combine the above-mentioned balance feature pyramid with the attention mechanism module, and the balanced semantic map $F_{b}$ features after balancing the multi-level feature map are refined and refined, respectively, through the channel attention mechanism and the spatial attention mechanism to obtain $F_{cab}$ and $F_{sab}$, as shown in Figure 5. Finally, the obtained feature map $F_{ab}$ is re-scaled and added with the original feature map to enhance multi-level features, ultimately improving the network feature extraction ability and improving the accuracy of the target detection model. The calculation expression is shown in Equation (6):

(6) $$F_{ab}=\left(M_{c}\left(F_{b}\right)⊗F_{b}\right)⊕\left(M_{s}\left(F_{b}\right)⊗F_{b}\right)$$

where $M_{c}$ and $M_{s}$ represent channel and spatial attention mechanism processing of balanced semantic feature map $F_{b}$. ⊗ represents the multiplication of corresponding elements, and ⊕ represents the addition of corresponding elements. The ABFP structure is shown in Figure 5.

The ABFP combines the balance feature pyramid and the attention mechanism to scale the multi-level features extracted from the backbone network of the detection model to different degrees, and then add an average to obtain the balanced semantic feature map. The balanced semantic feature map is input into the horizontal channel attention module and the spatial attention module, respectively, to obtain the channel attention balance semantic feature map and the spatial attention balance semantic feature map. The two are summed to obtain a refined balanced semantic feature map. Finally, the balanced semantic feature map is inversely scaled according to the resolution of different feature layers, and added to the original feature map to obtain the feature map enhanced by ABFP. The ABFP proposed in this paper can not only improve the unbalanced feature fusion problem of the detection model, but also combine with different feature fusion strategies to improve the accuracy of the detection model.

## 4. Experimental Results and Analysis

Two parts of experiments are designed according to the characteristics of the algorithm. On the one hand, the algorithm comparison experiment is carried out between different detection models, different feature fusion strategies, and different data sets. The other is the algorithm ablation experiment to prove the effectiveness of each module of the algorithm, including the BFP, channel attention mechanism module CA, and spatial attention mechanism module SA. The experimental results show that the ABFP method proposed in this paper can improve the traditional target detection methods, and can be compatible with different network models and feature fusion strategies.

### 4.1. Experimental Data and Environment

The data for this experiment are from 267 cases of SIST in the hospital, including 4993 labeled sections. The CT image file of each patient is a series of 3D image data stacked along the z-axis, which is obtained by scanning the patient’s abdomen layer by layer at 5 mm intervals with the corresponding equipment, and the data format is DICOM. The number of slices marked by doctors in each patient’s image sequence is about 2–18. We selected 229 patients’ image data as training and validation samples, including 4349 slices. The image data of 38 patients were used as test samples, including 644 slices.

This experiment was conducted on the Ubuntu 16.04LTS platform. The in-depth learning framework used is PyTorch framework, and Python language is used for code writing. The hardware configuration of this experiment is: Intel (R) Core i7-7800X CPU@3.50 GHz (Intel, Santa Clara, CA, USA), Nvidia RTX2080ti 11GB graphics card (Nvidia, Santa Clara, CA, USA), 64 GB running memory.

In the training Python model, the parameters used are: the input image size is 512 × 512, the number of input channels of FPN is [256, 512, 1024, 2048], the number of output channels is 256, the backbone network is ResNet101 network, the optimizer is SGD, the initial learning rate is 0.01, the learning momentum is 0.9, the weight attenuation is 0.0001, the training number is 24 epochs, the learning strategy is warmup linear learning strategy, the learning rate is reduced at the 16th and 22nd epochs, the batch_size is set to 4, and the image will flip left and right with a 50% probability level during training.

In this experiment, the RetinaNet model is used to train under the DeepLength [29] dataset, and the trained backbone network parameters are used as the pre-training model in the training of the SIST detection model, freezing the shallow network parameters and adjusting the deep parameters through training. Using the dataset of the same CT image as the pre-training model data can accelerate the convergence of the model and improve the detection performance of the model in the case of SIST data.

### 4.2. Comparative Experimental Analysis of Algorithms

In this section, we compared several sets of experiments to evaluate the effectiveness of the ABFP algorithm. These tests included comparing different models under the same feature fusion strategy, comparing the same model under different feature fusion strategies, and comparing results using the DeepLesion dataset.

This paper introduces two evaluation metrics for SIST target detection tasks: Average Precision (AP) and Average Recall (AR). Both of these metrics involve the calculation of Intersection over Union (IoU), which measures the degree of overlap between the model’s predicted regions and the doctor’s annotated regions. The calculation of IoU has a significant impact on the subsequent computation of AP and AR values.

Recall represents the proportion of positive instances, i.e., stromal tumors, correctly detected by the model out of all positive instances. It is primarily used to assess the model’s sensitivity to detecting stromal tumors. In this paper, the Average Recall (AR) is calculated by averaging recall values over multiple IoU thresholds, allowing for a better reflection of the model’s recall rate. This paper employs 10 IoU thresholds, specifically −0.50:0.05:0.95.

Average Precision (AP) is a comprehensive performance measure for object detection models and can be obtained by averaging the Precision-Recall (PR) curve.

Three different AP metrics are used in this paper: AP, AP75, and AP50. In the experimental results, AP and AR are similar, referring to the average of AP values across 10 IoU thresholds ranging from 0.50 to 0.95. This metric better reflects the model’s localization performance. AP75 and AP50, on the other hand, represent the AP values at IoU thresholds of 0.75 and 0.50, respectively, providing insight into the model’s localization capabilities. By using AP and AR, the paper conducts a comprehensive evaluation of the model’s localization and recall abilities in object detection.

**Figure 6:**
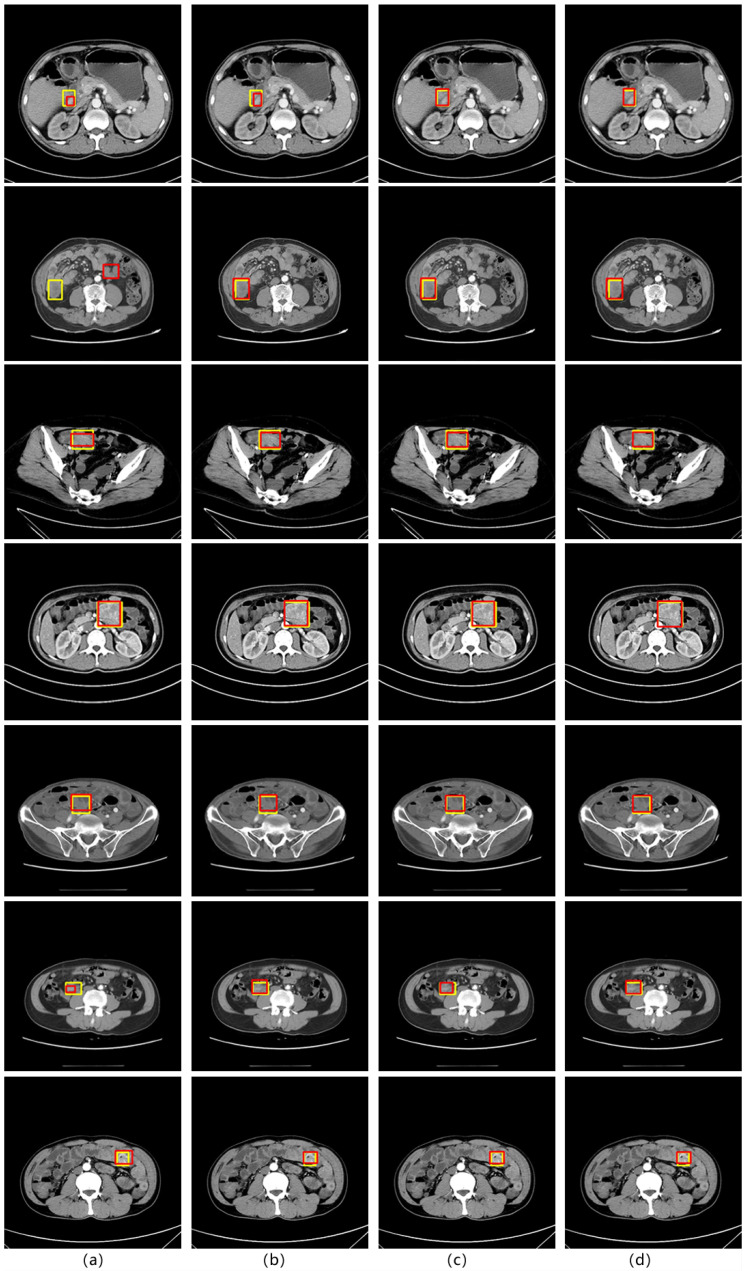
Comparison of test results of different models. ((**a**) is the test result of the original model itself, (**b**) is the result after adding the SE module, (**c**) is the result after adding the CBAM module, and (**d**) is the test result after adding the ABFP algorithm).

The first experiment is a comparative experiment under the same feature fusion strategy of different models. The results are shown in Table 1 and Figure 6. The experiment takes Faster RCNN and Mask RCNN as the baseline, and carries out comparative experiments on RetinaNet, RepPoints, and Cascade RCNN models, respectively. These three models, respectively, represent single-stage detection, two-stage detection, and “1.5”-stage detection framework with one classification and two regression, as well as the anchor base and anchor-free detection methods. Among them, the backbone network is the ResNet101 network, and the feature fusion strategy is FPN. In the comparison of the same model, it is compared with the mainstream attention mechanism SE attention mechanism, CBAM attention mechanism, and DCN, respectively. By combining with ResNet101 network with deformable convolutional DCN [30], it is proved that the method in this paper is compatible with networks with different structures.

In the small intestinal stromal tumor test set, ABFP can significantly improve the accuracy of the detection model whether in ResNet101 or in ResNet101 + DCN network, with DCN changing the network structure, as shown in Table 1. The ResNet101 + DCN network combined with the FPN + ABFP feature fusion strategy has achieved the best results in different models, with the test results reaching 0.614. In addition, in the comparative experiment, the AP values of the three different detection models increased from 0.526, 0.540, and 0.591 to 0.574, 0.574, and 0.614, respectively. In the same model structure, we can find that the improvement in the ABFP’s model detection ability on the basis of not changing the network model structure is close to the improvement in the mainstream attention mechanism SE, CBAM, and variable convolution DCN. In the RetinaNet and Cascade RCNN models, the improvement in ABFP on the model is between the SE attention mechanism and CBAM attention mechanism, while in the RepPoints model, the improvement in ABFP is slightly lower than SE and CBAM, and slightly higher than DCN.

The second experiment is a comparative experiment under different feature fusion strategies of the same model. The results are shown in Table 2. The experiment uses the RetinaNet model and ResNet101 network as the basis and carries out a comparative experiment with three different feature fusion strategies of FPN, PAFPN, and BIFPN, respectively. Comparing the test results of the model before and after adding the attention feature fusion pyramid ABFP proves the positive impact of ABFP on accuracy and compatibility with different feature fusion strategies. The input feature dimension of the three feature fusion methods is [256, 512, 1024, 2048], and the output feature dimension is 256.

In the small intestinal stromal tumor test set, the performance of the original PAFPN is better than that of FPN and BIFPN, which can be seen in Table 2. After adding the ABFP, the AP value of the RetinaNet detection model combined with the three feature fusion methods of FPN, BIFPN, and PAFAN increased from 0.526, 0.531, and 0.542 to 0.550, 0.562, and 0.559, respectively. For different feature fusion strategies, ABFP can combine well and improve the detection performance of the detection model, with good compatibility.

The third experiment is a comparative test conducted under the DeepLesion dataset. Due to the small size of the SIST dataset, the model proposed in this paper may have particularity. Therefore, the general applicability of the method proposed in this paper can be proved by testing and comparing in the open dataset DeepLesion, in which the training set is 35139 slices and the test set is 1616 slices. The results are shown in Table 3. In this group of experiments, the pre-training model uses an ImageNet data set to initialize the model and carries out comparative tests in RetinaNet, RepPoints, and Cascade RCNN models, respectively. The backbone network is ResNet101, and the feature fusion strategy is FPN. By comparing the performance of the three models before and after adding the ABFP, the ability of ABFP to improve the detection model in large data sets is proved.

From Table 3, compared with the original detection model, the test results of the model with the ABFP increased from 0.304, 0.345, and 0.318 to 0.338, 0.345, and 0.348, respectively, on the open dataset of DeepLesion. The experiment shows that in the large dataset of DeepLesion, the ABFP algorithm proposed in this paper can also better improve the model detection ability, and the improvement effect is more obvious because the dataset is large enough.

Through three different sets of comparative experiments, we observed that ABFP can better improve the performance of detection models of different structures under SIST data, and can be combined with different feature pyramid algorithms. The comparison diagram of test results is shown in Figure 6, which is represented by the Cascade RCNN network. The yellow rectangle in the diagram is the doctor’s mark, and the red rectangle is the model test result. In Figure 6, we can find that after the addition of the attention mechanism, the accuracy rate of stromal tumor detection is significantly improved. The detection results of the detection model without the addition of attention mechanism in the first and sixth lines are significantly different from the doctor’s label, especially in the second line, we can find that the model without the addition of attention mechanism has misdetection. The model with the addition of an attention mechanism can more accurately match the doctor’s label. In addition, we can find that for small stromal tumors, the ABFP algorithm proposed in this paper has better detection performance than SE and CBAM algorithms, and in the face of medium and large stromal tumors, the ABFP algorithm also better matches the doctor’s label.

### 4.3. Ablation Experiment of Attention Balance Characteristic Pyramid (ABFP)

The ABFP model proposed in this section uses the BFP, the channel attention module, and the spatial attention module, and combines the balanced semantic features of SIST, the channel dimension features, and the spatial region features of SIST to detect lesions. In order to verify the effectiveness of different modules and improve the detection model, the ABFP model will be ablated in this experiment. First, the baseline is the test result of the RetinaNet model without adding ABFP, the backbone network is ResNet101, and the feature fusion method is FPN. Then, only the BFP is added, in which the refinement operation is replaced by a convolution operation, and then the channel attention mechanism module is added first, and then the spatial attention mechanism module is added for comparative test. The experimental results are shown in Table 4.

As can be seen from Table 4, after adding only the BFP, the accuracy of the detection model was increased from 0.526 to 0.529. After adding the channel attention mechanism module to refine the balanced semantic features, the accuracy of the detection model was increased from 0.529 to 0.539. After adding the spatial attention mechanism module, the accuracy of the detection model was increased from 0.539 to 0.550. The experimental results show that the BFP processing of multi-level features is effective, and the detection model is improved by refining and improving the channel dimension and spatial dimension of the balanced semantic features and then fusing them.

## 5. Conclusions

Aiming at the problem that the mainstream feature fusion methods on the SIST data set do not deal with the deep semantic feature and the shallow detailed unbalanced feature fusion well, an SIST algorithm based on the ABFP is proposed. In this algorithm, firstly, the multi-level feature map extracted from the detection model backbone network is rescaled to the same size as the middle layer, and all the scaled feature maps are weighted and fused to obtain a balanced semantic feature map. Then, the channel attention module and the spatial attention module are introduced to refine the balanced semantic feature map in the channel dimension and the spatial dimension, respectively, and the balanced semantic feature map containing more feature information is obtained. Finally, the balanced semantic feature map is re-scaled back to the size of each level feature map and added to the original feature map of each level to enhance the original features. The ABFP algorithm can improve the accuracy of the detection model.

However, this approach is constrained by the size of the stromal tumor dataset in practical applications, and this issue warrants further consideration. It necessitates larger datasets to support model training and validation, enhancing its generalization and robustness. Challenges related to data scale also involve the complexity and cost of data annotation. Therefore, effectively expanding and enriching the dataset presents a significant challenge for our future research endeavors.

In real-world diagnostic assistance, the issue of false positive rates in the model still remains a pressing concern. Striking a balance between reducing false alarms while maintaining high sensitivity is a formidable task. We need to explore more intelligent algorithms and strategies to further enhance the model’s precision and reliability, making it more applicable in medical practice.

Furthermore, the algorithms designed in this paper and the detection models employed are primarily situated in the domain of two-dimensional image processing, overlooking the contextual information between different slices in medical image sequences. This means that we have overlooked the spatiotemporal correlation, which is typically crucial in medical image analysis. Therefore, the next phase of research may need to focus on extending our current algorithms and models to handle three-dimensional images, enabling a more accurate capture of lesion location and morphological characteristics.

In conclusion, while the current approach exhibits promise, we must recognize the challenges associated with dataset limitations, false positives, and the transition to three-dimensional image analysis. By addressing these issues, we can better advance research in the field of medical image analysis, ultimately providing more reliable and efficient tools for medical practice. 

## Figures and Tables

**Table 1:** Comparative experimental results of different detection models (Bold indicates best performance).

Model	Backbone	Neck	AP	AP0.75	AP0.50	AR
Faster RCNN [25]	ResNet 101	FPN	0.547	0.636	0.866	0.615
Mask RCNN [31]	ResNet 101	FPN	0.556	0.642	0.847	0.624
RetinaNet [26]	ResNet 101	FPN	0.526	0.597	0.846	0.619
	ResNet 101+SE	FPN	0.551	0.634	0.853	0.640
	ResNet 101 + CBAM	FPN	0.549	0.596	0.891	0.648
	ResNet101 + DCN	FPN	0.553	0.632	0.873	0.638
	ResNet 101	FPN + ABFP	0.550	0.612	0.886	0.651
	ResNet 101 + DCN	FPN + ABFP	0.574	0.662	0.900	0.662
RepPoints [28]	ResNet 101	FPN	0.540	0.584	0.885	0.624
	ResNet 101 + SE	FPN	0.554	0.628	0.885	0.637
	ResNet 101 + CBAM	FPN	0.560	0.620	0.906	0.647
	ResNet 101 + DCN	FPN	0.549	0.594	0.886	0.638
	ResNet 101	FPN + ABFP	0.550	0.604	0.882	0.669
	ResNet 101 + DCN	FPN + ABFP	0.574	0.633	0.924	0.649
Cascade RCNN [27]	ResNet 101	FPN	0.591	0.694	0.847	0.652
	ResNet 101 + SE	FPN	0.598	0.708	0.862	0.659
	ResNet 101 + CBAM	FPN	0.610	0.713	0.898	0.682
	ResNet 101 + DCN	FPN	0.601	0.703	0.855	0.663
	ResNet 101	FPN + ABFP	0.604	0.706	0.907	0.678
	ResNet 101 + DCN	FPN + ABFP	**0.614**	**0.731**	**0.920**	**0.687**

**Table 2:** Comparison test results of different feature fusion strategies (Bold indicates best performance).

Model	Neck	AP	AP0.75	AP0.50	AR
Retinanet + ResNet101	FPN	0.526	0.597	0.846	0.619
	FPN + ABFP	0.550	0.612	0.886	0.651
	BIFPN	0.531	0.615	0.845	0.624
	BIFPN + ABFP	0.562	0.643	0.908	0.668
	PAFPN	0.542	0.618	0.880	0.647
	PAFPN + ABFP	**0.559**	**0.622**	**0.925**	**0.663**

**Table 3:** Comparison test results under DeepLesion dataset (Bold indicates best performance).

Model	Neck	AP	AP0.75	AP0.50	AR
RetinaNet	FPN	0.304	0.337	0.519	0.579
	FPN + ABFP	0.338	0.379	0.570	0.584
RepPoints	FPN	0.305	0.340	0.517	0.585
	FPN + ABFP	0.345	0.387	0.587	0.599
Cascade RCNN	FPN	0.318	0.363	0.539	0.537
	FPN + ABFP	**0.348**	**0.400**	**0.573**	**0.544**

**Table 4:** Results of ABFP ablation experiment (Bold indicates best performance).

Model	BFP	CA	SA	AP	AP0.75	AP0.50	AR
ABFP				0.526	0.597	0.846	0.619
	*√*			0.529	0.601	0.856	0.630
	*√*	*√*		0.539	0.590	0.874	0.639
	*√*	*√*	*√*	**0.550**	**0.612**	**0.886**	**0.651**

## Data Availability

The datasets generated or analyzed during this study are available from the corresponding author on reasonable request.
